# PD-L1 expressing circulating tumour cells in head and neck cancers

**DOI:** 10.1186/s12885-017-3316-3

**Published:** 2017-05-16

**Authors:** Arutha Kulasinghe, Chris Perry, Liz Kenny, Majid E. Warkiani, Colleen Nelson, Chamindie Punyadeera

**Affiliations:** 1 The School of Biomedical Sciences, Institute of Health and Biomedical Innovation, Queensland University of Technology, Kelvin Grove, QLD Australia; 2 Translational Research Institute, Brisbane, Australia; 3 Department of Otolaryngology, Princess Alexandra Hospital, QLD, Woolloongabba, Australia; 4 School of Medicine, University of Queensland, Brisbane, QLD Australia; 5 Royal Brisbane and Women’s Hospital, Brisbane, QLD Australia; 6 Central Integrated Regional Cancer Service, Queensland Health, Brisbane, QLD Australia; 7 School of Mechanical and Manufacturing Engineering, Australian Centre for NanoMedicine, University of New South Wales, Sydney, Australia; 8 Garvan Institute for Biomedical Research, Sydney, Australia; 9 School of Medical Sciences, Edith Cowan University, Joondalup, Perth, WA 6027 Australia; 10 Australian Prostate Cancer Research Centre - Queensland, Institute of Health and Biomedical Innovation, Queensland University of Technology, Princess Alexandra Hospital, Translational Research Institute Brisbane, Brisbane, Australia

**Keywords:** PD-L1, Head and neck cancers, Circulating tumour cells, Non-invasive tools, Liquid biopsy

## Abstract

**Background:**

Blockade of the PD-1/PD-L1 immune checkpoint pathway is emerging as a promising immunotherapeutic approach for the management and treatment of head and neck cancer patients who do not respond to 1st/2nd line therapy. However, as checkpoint inhibitors are cost intensive, identifying patients who would most likely benefit from anti PD-L1 therapy is required. Developing a non-invasive technique would be of major benefit to the patient and to the health care system.

**Case presentation:**

We report the case of a 56 year old man affected by a supraglottic squamous cell carcinoma (SCC). A CT scan showed a 20 mm right jugulodigastric node and suspicious lung lesions. The lung lesion was biopsied and confirmed to be consistent with SCC. The patient was offered palliative chemotherapy. At the time of presentation, a blood sample was taken for circulating tumour cell (CTC) analysis. The dissemination of cancer was confirmed by the detection of CTCs in the peripheral blood of the patient, measured by the CellSearch System (Janssen Diagnostics). Using marker-independent, low-shear spiral microfluidic technology combined with immunocytochemistry, CTC clusters were found in this patient at the same time point, expressing PD-L1.

**Conclusion:**

This report highlights the potential use of CTCs to identify patients which might respond to anti PD-L1 therapy.

**Electronic supplementary material:**

The online version of this article (doi:10.1186/s12885-017-3316-3) contains supplementary material, which is available to authorized users.

## Background

Head and neck cancer (HNC) patients often present with advanced metastatic disease. Whilst there have been improvements in the management of locoregional disease, distant metastatic spread remains a challenge in the field [1–3]. Palliative chemotherapy is platinum based and for patients who progress after first line treatment or are refractory, therapeutic options are limited. Numerous agents including cetuximab, paclitaxel, gemcitabine and docetaxel have been assessed prospectively in the treatment of platinum refractory patients and the time to progression ranged from 2 to 6 months [4]. These systemic treatments produce a significant degree of morbidity and new therapeutic options are therefore a need in these patients. Once there is an established role in metastatic disease, translation into the curative setting is appropriate.

The programmed cell death-1/programmed cell death-1 ligand (PD-1/PD-L1) pathway has shown to play a crucial role in tumour immune invasion. Recent literature suggests that PD-L1 over expression in solid tumour types has shown direct tumour protection. Recent studies have shown that antibodies targeting PD-1/PD-L1 have significant anti-tumour activity with a much lower toxicity profile and are currently being investigated in a number of tumour types [5, 6]. Pembrolizumab (previously MK-3475) is a highly selective, humanized igG4 (kappa) isotype monoclonal antibody designed to block PD-1 interacting with ligands, PD-L1 and PD-L2, thereby allowing the immune system to target and destroy the tumour. Pembrolizumab was the first anti PD-1 antibody to be approved by the FDA [6].

In the 2014, American Society of Clinical Oncology (ASCO) meeting, it was reported that in a majority (77.9%) of pre-treated HNSCC patients, PD-L1 is expressed in the tumour, defined by ≥1% stained cells in the tumour microenvironment [7]. In the Keynote 012 trial presented at ASCO 2015, tumour shrinkage was found in 57% of patients, and overall response of 24.8%, comprised of 26.3% in HPV-negative and 20.6% HPV-positive patients [5, 8]. The Keynote 012 study indicated that Pembrolizumab was twice as effective as cetuximab with durable responses in patients which has not been seen previously in HNC. Pembrolizumab was also well tolerated in these patients with low rates of adverse effects. 86% of the responsive patients enrolled in the Keynote 012 study continued to receive treatment highlighting the acceptable safety profile [7, 8].

Metastatic sites have shown unique genomic alterations, which can be quite different from the primary site [9, 10]. Invasive procedures are currently required to biopsy these metastatic sites, some of which may be inaccessible. Other studies have shown that these biopsies may not be representative of all of the metastatic disease [11]. An alternative approach used in other cancer types is the analysis of blood samples for circulating tumour cells (CTCs) as a form of “liquid biopsy” [12–14]. These rare tumour cells in circulation represent the “transient” cancer cell population that have the propensity to metastasize to distant sites. Recent reports have shown how CTCs may provide complementary information to identify candidate therapeutic targets and drug resistance mechanisms [9, 12, 15]. Moreover, CTCs represent cells from the primary and metastatic sites, thereby possibly providing a more comprehensive overview of the tumour burden of an individual patient. CTCs in the blood of HNC patients provide an opportunity to identify patients “at-risk” of developing overt metastasis in due course. More importantly, the analysis of these metastatic seeds in circulation may reveal important information for systemic therapy targeting metastatic disease [9]. CTCs are currently being investigated as predictive biomarkers for HER-2 targeted therapies [16]. A similar strategy could be used for immune checkpoint blockade therapies such as PD-L1.

We report the case study of a 56 year old man who had been diagnosed with a supraglottic SCC and treated with chemoradiotherapy. The patient was assessed for CTCs by the FDA-approved CellSearch (Janssen Diagnostics) and spiral microfluidics platform. Single CTCs were detected by both platforms. Using the spiral approach, CTC clusters were identified expressing PD-L1. We propose the notion that CTC PD-L1 assessment may be an avenue to identify patients who would be suitable candidates for anti-PDL1 therapy.

## Case presentation

A 56 year old Caucasian male with a background history of Crohn’s disease, treated with azathioprine, presented at the end of 2013 with a supraglottic T3N2b squamous cell carcinoma which was treated with upfront chemoradiotherapy, utilising Cisplatin. In October 2014, a CT scan showed a 20 mm right jugulodigastric node. Lung lesions were observed which were queried to be fungal/distant metastasis by the MDT clinic. Biopsied specimen of the lung lesions confirmed moderately differentiated SCC. Progressive disease to the lungs and pelvic bone were observed whilst on Taxol and Carboplatin, given weekly. Having limited options, a trial of infusional 5-FU (fluorouracil) was given at the end of 2015. The patient died in February 2016.

### Chest X-ray

**Fig. 1:**
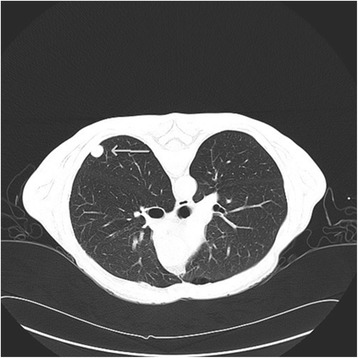
Chest CT scan of Patient showing lung lesion indicated by arrow. The lung biopsy was consistent with moderately differentiated SCC

Nodules were projected over the left infrahilar region and within the right upper lobe suspicious for SCC metastasis (Fig. 1).

### CTC assessment by CellSearch

**Fig. 2:**
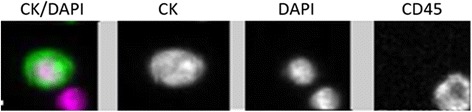
Patient presented with CellSearch-positive CTCs in circulation at time of presentation to clinic. CTCs: EpCAM + CK + DAPI + CD45-, Leukocytes: CD45 + DAPI+

The Patient presented with CellSearch-positive CTCs (2CTCs/7.5 ml) in circulation at time of presentation to clinic (Fig. 2).

### CTC assessment by spiral technology

**Fig. 3:**
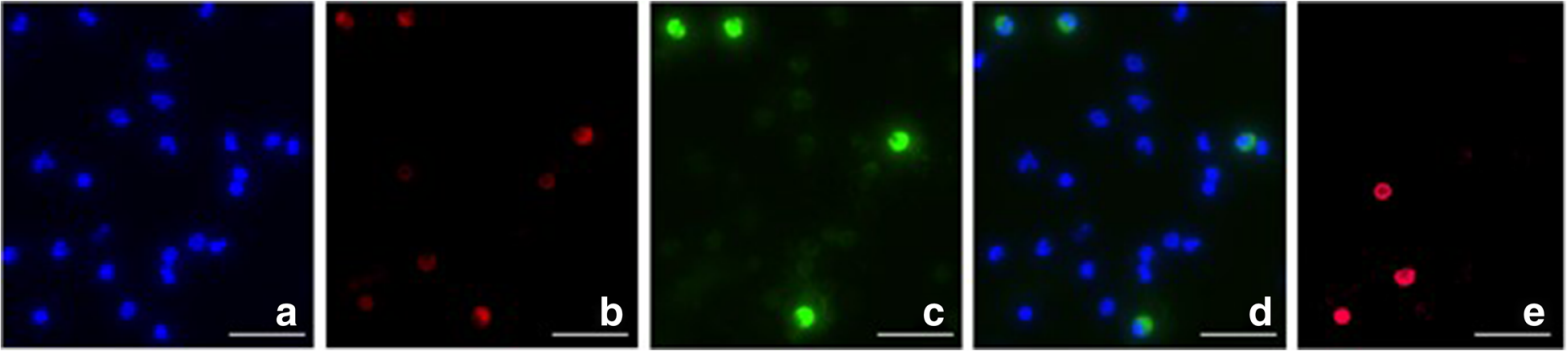
Single CTCs detected after enrichment using spiral microfluidics. Immunofluorescent staining for (**a**) DAPI (**b**) Cytokeratin (**c**) EGFR (**d**) Composite EGFR/DAPI (**e**) CD45. CTCs: EGFR + CK + DAPI + CD45-. White blood cells: CD45 + DAPI+. Scale bar represents 50 μm

**Fig. 4:**
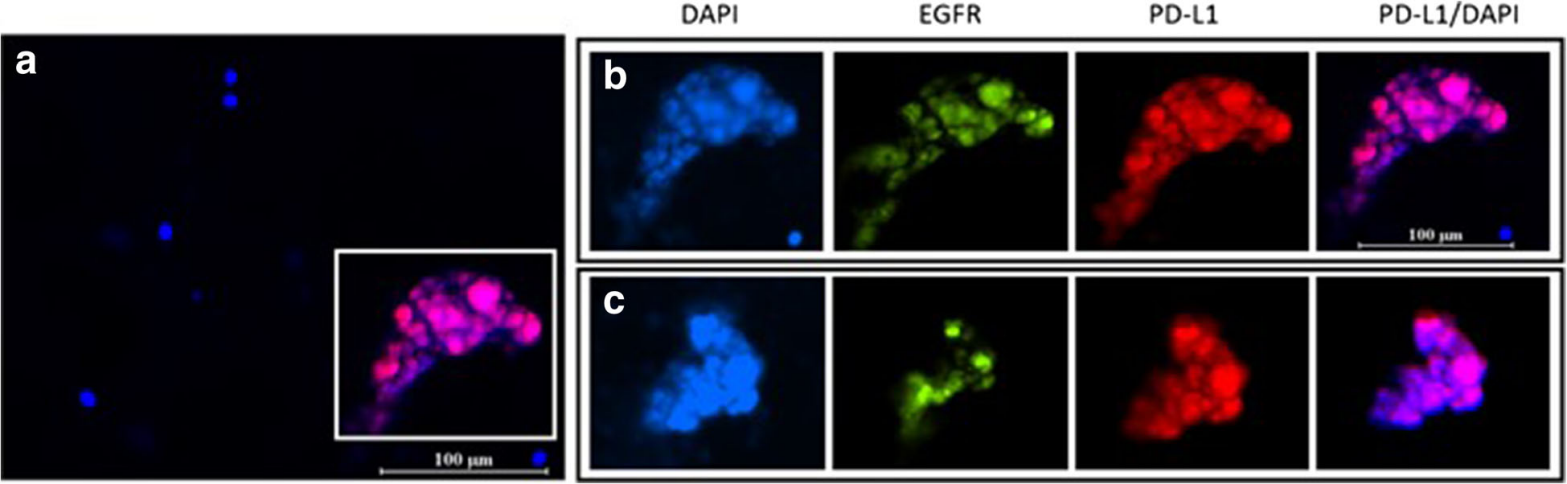
CTC clusters detected after enrichment using spiral microfluidics. **a** 200X magnification composite PD-L1/DAPI (**b**) CTC cluster magnified (1000X) showing individual and composite images for DAPI, EGFR, PD-L1 and PD-L1/DAPI. **c** 1000X magnification of a further CTC cluster present in the same patient characterized for the same cellular markers. Scale bar represents 100 μm

**Fig. 5:**
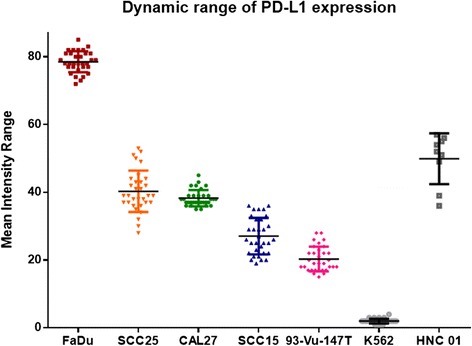
Range of expression of PD-L1 across 5 HNC cell lines (FaDu, SCC25, CAL27, SCC15, 93-Vu-147 T), negative control (K562) and patient sample (HNC01)

The Patient presented with 4 single CTCs (EGFR + CK + DAPI + CD45-) (Fig. 3) and 2 CTC clusters (EGFR + PDL1 + DAPI+) (Fig. 4). The CTC clusters showed a PD-L1 mean intensity in the mid to high dynamic range, determined using a panel of known HNC cell lines (Fig. 5).

### Immunoassay

The antibody against PD-L1 showed a relatively high mean intensity for (FaDu-Additional file 1: Fig. S1), medium (SCC25, CAL27), low (SCC15, 93-VU-147 T) and a negative control (K562 – Additional file 1: Fig. S2). PD-L1 staining of the CTC channel with spiked SCC-15 cells (Additional file 1: Fig. S3) and waste channel showing the bulk of leukocytes (Additional file 1: Fig. S4).

### Isolation of CTCs by CellSearch

7.5 mL of whole blood collected in CellSave blood collection tubes (Janssen Diagnostics) was mixed with 6.5 ml of CellTracks™ buffer and centrifuged at 800 x g for 10 mins. The sample was placed on the AutoPrep™ system and the protocol followed as per manufacturer’s instructions. The system uses a positive selection enrichment based on EpCAM antibodies and characterizes the selected cells for pan-cytokeratin (pan-CK), CD45 and DAPI staining. A CTC was determined based on the following parameters: positive for EpCAM, pan-CK, DAPI, at least a diameter of 4x4μm and negative for CD45 (common leukocyte marker). The results were reported as the number of CTCs/7.5 ml whole blood.

### Enrichment of CTCs by spiral microfluidics technology

8 mL of whole blood was collected in EDTA tubes (BD-Plymouth, UK). To reduce the cellular components passing through the spiral chip, a red blood cell (RBC) lysis was performed. Briefly, post RBC lysis (Astral Scientifix), the sample was centrifuged and the pellet resuspended in 10 mL of sheath buffer (1xPBS, 2 mM EDTA, 0.5% BSA). Tygon® tubing was inserted into the inlet/outlets of the spiral chip, and the inlet tubing connected to a syringe pump. The spiral chip was fixed into position on a phase contrast microscope (Olympus, IX71). The outlet tubing was connected to sterile 15 mL BD falcon collection tubes. To run the patient sample, the sample was carefully loaded into a 10 ml syringe and pumped through the spiral chip at a flow rate of 1.7 ml/min [17]. The outputs were collected and spun down at 300 x g for 5 mins. The enriched cell suspension was cytospun onto 2 glass slides using the Cytospin™ 4 Cytocentrifuge (ThermoScientific, USA). The presence of CTCs was determined by immunofluorescent staining.

### Development of an immunoassay for PD-L1

Five head and neck cell lines were used to develop a dynamic range of PD-L1 expression. FaDu (ATCC®HTB43™), CAL27 (ATCC®CRL2095™), SCC25 (ATCC®CRL-1628™) were from the American Type Culture Collection (ATCC™). SCC15 (ATCC®CRL-1623™) a generous gift from Dr. Glen Boyle (QIMR, Brisbane) and 93-VU-147 T (CVCL_L895) (HPV-positive) cell line from Dr. Johan de Winter (VU Medical Center, Netherlands). The human chronic myelogenous leukemia K562 (ATCC®CCL-243) cells were used as a negative control (gift from Prof Maher Gandhi, UQDI, Brisbane). Briefly, cytospins (Cytospin™ Cytocentrifuge, USA) were prepared using aliquots of 1000 cells/slide by centrifugation at 300 x g for 5 mins. The slides were stained with PD-L1 as per the immunocytochemistry protocol below.

### Immunocytochemistry

Briefly, the initial sample was stained using the CellSearch antibody cocktail (Janssen Diagnostics) and anti-EGFR antibody (AY13, Biolegend, San Diego) as previously described [18–20]. A further slide was fixed with 4% formaldehyde for 10 mins, permeabilized with 0.2% Triton X-100 for 5 mins and blocked with 10% fetal-bovine serum in 0.1% PBS-Tween for 1 h at room temperature. The cells were incubated overnight at 4 °C with anti-EGFR antibody and anti-PD-L1 antibody [28-8] (Alexa Fluor® 647) (Abcam ab209960) 1/100 dilution. Nuclear DNA was visualized with DAPI. Rabbit IgG monoclonal isotype control (Alexa Fluor®647) (ab199093) was used to identify nonspecific binding. Cells were imaged on the Olympus IX3 inverted microscope.

## Discussion and conclusions

The predictive value of PD-L1 expression in primary tissues is limited [9]. Furthermore, there is weak correlation between matched primary tumour and distant metastasis, suggesting that primary tumour is not an adequate surrogate for determining PD-L1 expression at metastatic sites [21]. Importantly, this highlights the fact that a single core biopsy may not suffice to determine tumour PD-L1 expression [9]. The FDA-approved CellSearch technology has shown clinical significance of EpCAM-positive CTCs [22]. However, there has been a shift in the field to maker-independent technologies to capture a greater proportion of CTCs in circulation in an unbiased fashion [18, 23]. By the use of established [9, 22] and spiral microfluidics technology [17, 24] this study aimed to capture the tumour cells in circulation of this patient and characterize the PD-L1 expression.

The patient presented with CTCs by enrichment using both CellSearch and spiral technologies. Importantly, the spiral technology was able to enrich for CTC clusters which are rarely detectable by CellSearch [25]. These microemboli/tumour cell clusters have shown an increased metastatic potential compared to single cells [26, 27]. Moreover, the cluster had a mid-high PD-L1 expression compared to the panel of known head and neck cancer cell lines. The PD-L1 + CTCs indicate that the patient had tumour cells in circulation with the capacity to block the immune system. These could be a potential targets for PD-L1 therapies [9]. The development of an immune-score is desirable for metastatic HNC patients.

In the 2016 AACR meeting, in locally advanced HNC patients, PD-L1+ CTCs were associated with shorter progression free survival (PFS) and overall survival (OS), and were proposed to “select and monitor patients for PD-1 checkpoint inhibitors” [28, 29]. These studies as well as this study, demonstrate that PD-L1 is expressed in HNSCC tumours and CTCs and may contribute to the tumours ability to evade the immune system. Moreover, that PD-L1 may be used as a biomarker for predicting responders from non-responders in lieu of the cost burden of such therapies.

This case report highlights the potential of using of CTCs to (i) identify patients ‘at-risk’ of developing metastasis (ii) identifying HNC patients that are likely to benefit from anti PD-L1 therapy and (iii) the development of a CTC PD-L1 immune-score for HNC. Further studies are warranted comparing patient tumour and CTC PD-L1 expression to develop predictive biomarkers.

## Additional file

Additional file 1: Fig. S1. HNSCC cell lines (FaDu) immunofluorescent staining with DAPI (Blue), PD-L1 (Red). Scale bar represents 10 μm. **Fig. S2** Rabbit IgG monoclonal Isotype control (AlexaFluor 647) (ab199093). Scale bar represents 10 μm. **Fig. S3** DAPI and PD-L1 staining on HNSCC cell line (SCC15) spiked into Normal healthy blood and sorted on the spiral chip (CTC outlet). White arrows indicate leukocytes in the background of spiked tumour cells. Scale bar represents 10 μm. **Fig. S4** PD-L1 staining of white blood cells in the waste channel of the spiral chip. Scale bar represents 10 μm. (DOCX 1176 kb)

## Abbreviations

5-FU: 5-fluorouracil
BSA: Bovine serum albumin
CK: cytokeratin
CTC: circulating tumour cells
DAPI: 4′,6-diamudubi-2-phenylindole
EGFR: epidermal growth factor recptor
FDA: Food and Drug Administration
HNC: Head and neck cancer
NHMRC: National Health and Medical Research Council
OS: Overall Survival
PAH: Princess Alexandra Hospital
PBS: Phosphate buffered saline
PD-L: Programmed cell death ligand
PD-L1: Programmed cell death ligand one
PFS: Progression free surivival
SCC: squamous cell carcinoma
UQ: University of Queensland
