# Supplementary material for: PD-L1 expressing circulating tumour cells in head and neck cancers
Source: BMC Cancer. 2017 May 16;17:333. doi: 10.1186/s12885-017-3316-3 (PMC5434641; doi:10.1186/s12885-017-3316-3)
Supplement: Additional file 1: Fig. S1. — HNSCC cell lines (FaDu) immunofluorescent staining with DAPI (Blue), PD-L1 (Red). Scale bar represents 10 μm. Fig. S2 Rabbit IgG monoclonal Isotype control (AlexaFluor 647) (ab199093). Scale bar represents 10 μm. Fig. S3 DAPI and PD-L1 staining on HNSCC cell line (SCC15) spiked into Normal healthy blood and sorted on the spiral chip (CTC outlet). White arrows indicate leukocytes in the background of spiked tumour cells. Scale bar represents 10 μm. Fig. S4 PD-L1 staining of white blood cells in the waste channel of the spiral chip. Scale bar represents 10 μm. (DOCX 1176 kb) [file 12885_2017_3316_MOESM1_ESM.docx]

Additional file 1


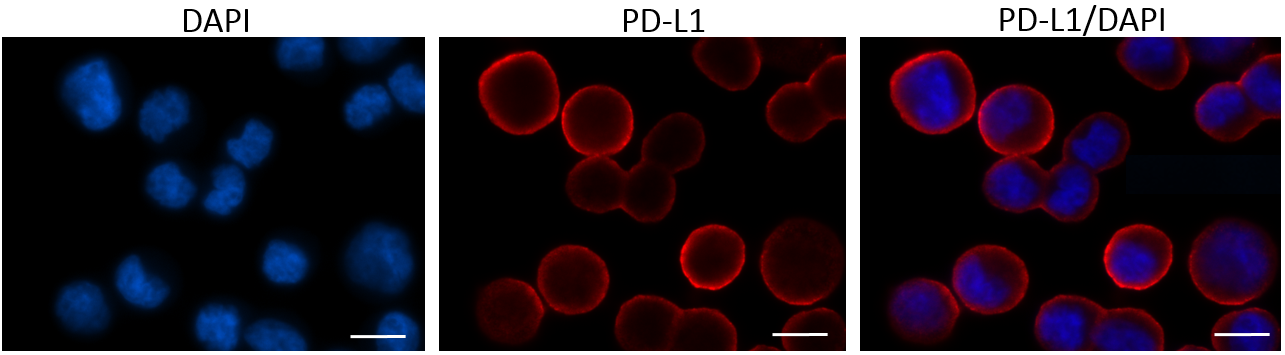


Figure S1. HNSCC cell lines (FaDu) immunofluorescent staining with DAPI (Blue), PD-L1 (Red). Scale bar represents 10 µm.

DAPI PD-L1


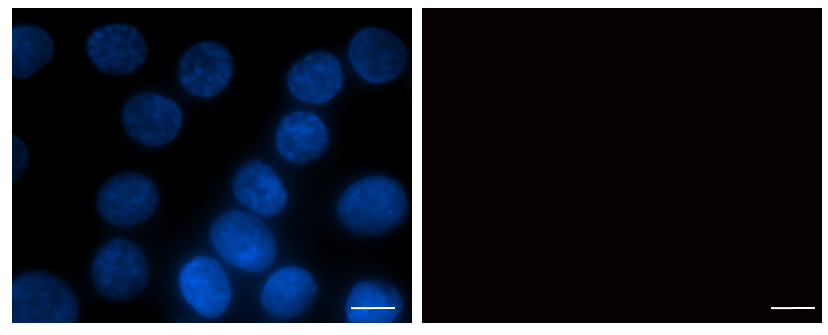


Figure S2. Rabbit IgG monoclonal Isotype control (AlexaFluor 647) (ab199093). Scale bar represents 10 µm.


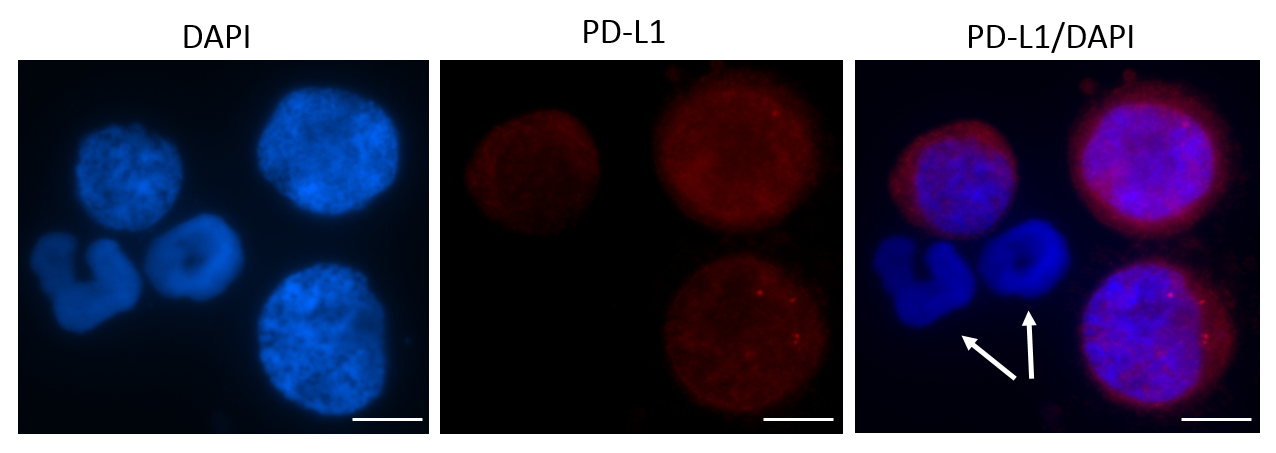


Figure S3. DAPI and PD-L1 staining on HNSCC cell line (SCC15) spiked into Normal healthy blood and sorted on the spiral chip (CTC outlet). White arrows indicate leukocytes in the background of spiked tumour cells. Scale bar represents 10 µm.


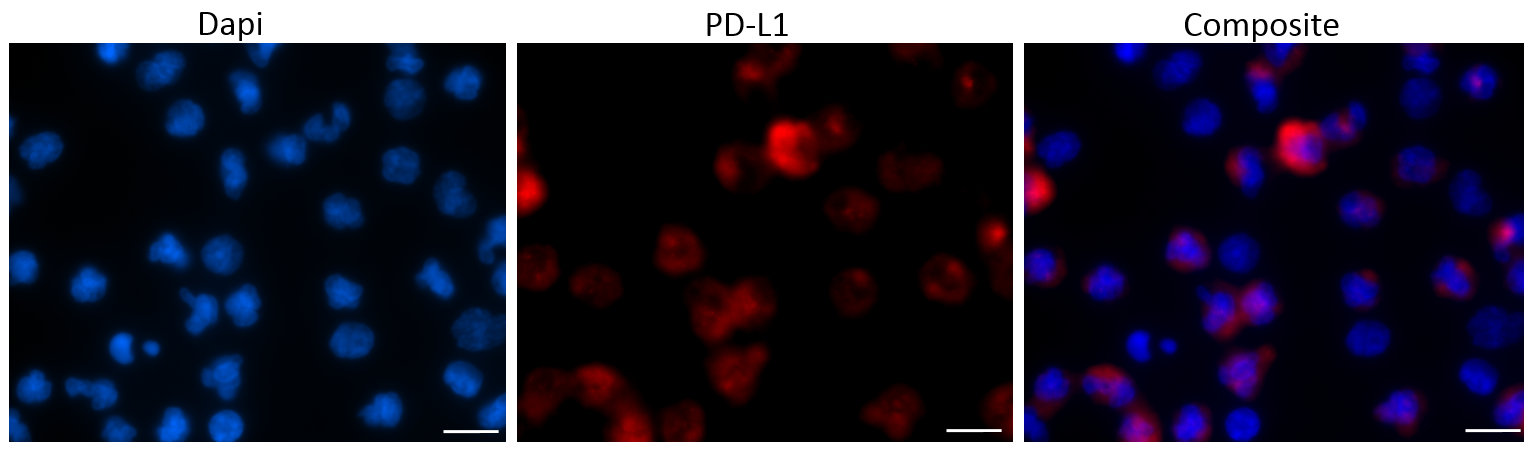


Figure S4. PD-L1 staining of white blood cells in the waste channel of the spiral chip. Scale bar represents 10 µm.
